# First autochthonous cases of canine thelaziosis in Slovakia: a new affected area in Central Europe

**DOI:** 10.1186/s13071-017-2128-2

**Published:** 2017-04-13

**Authors:** Viktória Čabanová, Peter Kocák, Bronislava Víchová, Martina Miterpáková

**Affiliations:** 1 Institute of Parasitology, Slovak Academy of Sciences, Hlinkova 3, 040 01 Košice, Slovakia; 2 Private Veterinary Practice, Pekárenská 1, 071 01 Michalovce, Slovakia

**Keywords:** Canine thelaziosis, *Thelazia callipaeda*, Dogs, Zoonoses, Central Europe, Vector-borne diseases

## Abstract

**Background:**

The spirurid nematode *Thelazia callipaeda*, also called the “Oriental eyeworm”, is the causative agent of canine and human ocular thelaziosis. In the past few years it has started to spread across central Europe and new endemic areas have been established. The present study reports on the first four autochthonous cases of canine ocular thelaziosis in the territory of Slovakia, Central Europe.

**Results:**

All cases were recorded in dogs living in eastern Slovakia, near the border with the Ukraine. All worms collected were investigated morphologically and their identification further confirmed at the molecular level by PCR amplification and direct sequencing. Nucleotide sequences of partial *T. callipaeda cox*1 and 28S rDNA gene fragments isolated from Slovak dogs were submitted to the GenBank database under accession numbers KY476400 and KY476401, respectively.

**Conclusions:**

Considering that all four cases were diagnosed in animals that had never travelled abroad, there is clear evidence of an autochthonous occurrence and thereby the further spread of *T. callipaeda* across Europe. Moreover, at latitude of 48°N, these cases might be considered as the northernmost recorded cases of autochthonous in western and Central Europe.

## Background

The spirurid nematode *Thelazia callipaeda* Railliet & Henry, 1910, also called the “Oriental eyeworm”, is the causative agent of ocular thelaziosis. The parasite was isolated for the first time from a dog in Pakistan in 1910. In 1917, the first human cases were confirmed in China, and the zoonotic character of the eyeworms was revealed [1]. *Thelazia callipaeda* is found in the conjunctival sacs and nictitating membranes of humans and both domestic and wild-living carnivores. The most common clinical manifestations are conjunctivitis, lacrimation and itchiness, but the infection can sporadically lead even to blindness [1]. As has been demonstrated in several studies, the fruit fly *Phortica variegata* (Diptera: Drosophilidae) seems to be the main vector of the eyeworms responsible for canine thelaziosis in Europe [2, 3].

The first documented cases of canine thelaziosis came from north-western Italy, when *T. callipaeda* was detected in 16 dogs from the Piedmont region [4]. After 20 years, infection was also recorded in other countries of western Europe: in France [5], Switzerland [6], Germany [7] and Belgium [8]. Most recently autochthonous thelaziosis was observed in dogs from the Balkan area: 27 cases of infected dogs were recorded in the region of Sofia, Bulgaria [9]; the spread of canine thelaziosis was described in Croatia and Bosnia and Herzegovina [10]; and *T. callipaeda* was recorded in dogs for the first time in 2012 and 2013 in Serbia and in 2014 in Romania [11, 12]. Recently, autochthonous cases of thelaziosis were described in dogs, cats and even in one rabbit from northern and central regions of Greece [13]. In 2016 the first human cases of thelaziosis were also reported from Croatia and Serbia [14, 15].

However, to our knowledge, no data on autochthonous canine thelaziosis are known from the region of Central Europe, including Austria, Czech Republic, Ukraine, Poland and Slovakia. Nevertheless, *T. callipaeda* has now for the first time been recorded in one dog from Hungary [16]. Furthermore, the ecological niche model [17] showed that central Europe (including all of the above-mentioned countries) is suitable for the establishment of the vector *P. variegata*.

In this study we present the first cases of canine thelaziosis in dogs from Slovakia.

## Methods

### Clinical case reports

**Fig. 1:**
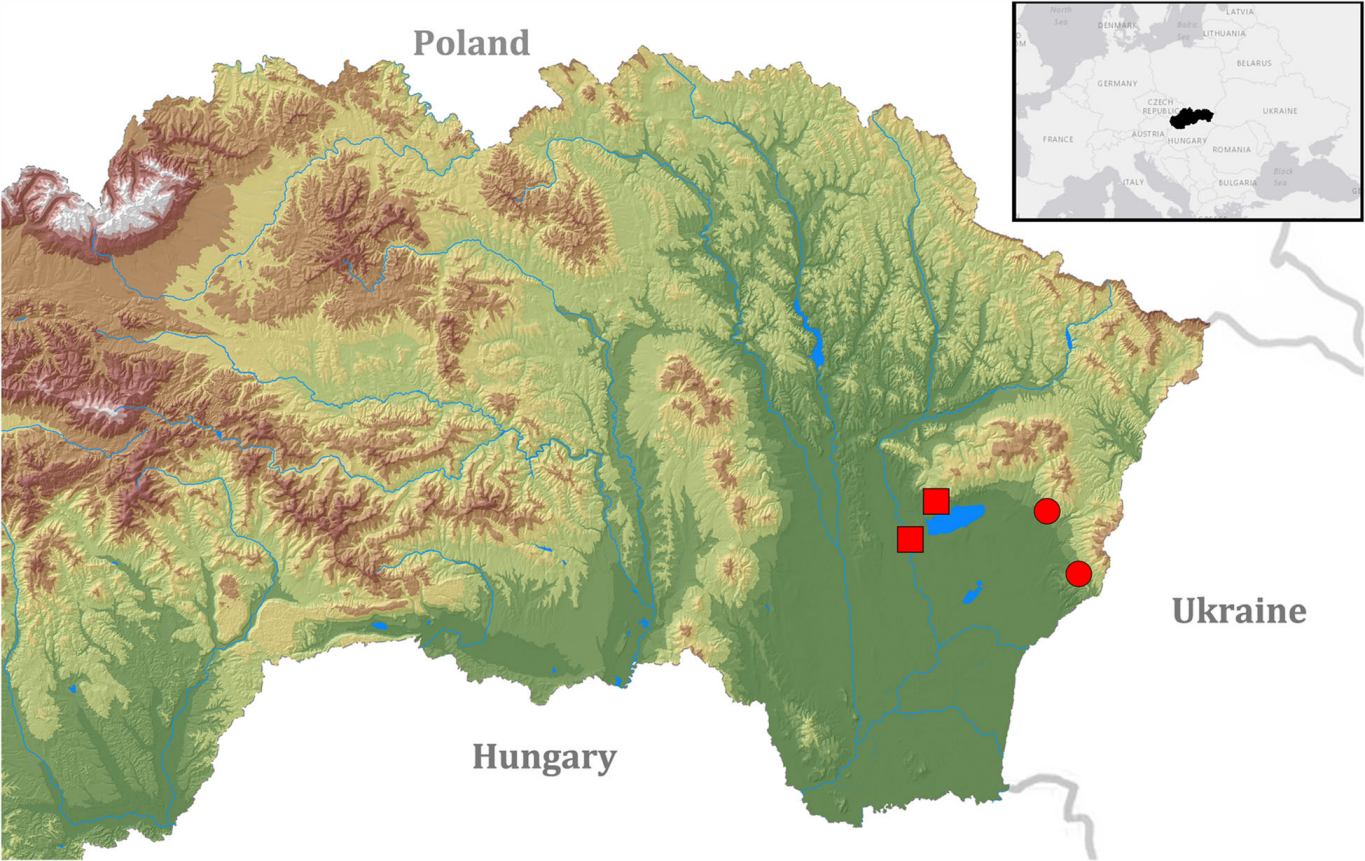
Map of localities for dogs infected by *Thelazia callipaeda* in Slovakia. Circles represent two cases of dog thelaziosis from villages of Koromľa and Hlivištia (September 2016) and squares represent two other cases from Michalovce and Vinné (October 2016)

During autumn 2016, four autochthonous cases of canine thelaziosis were recorded in Slovakia within a 6-week period. The first two cases were diagnosed in September 2016 in Sobrance district, eastern Slovakia, close to border with the Ukraine (Fig. 1). In the first case, an 18-month-old female dog of the breed Large Münsterländer from Koromľa (48°42'57"N, 22°17'38"E) without any travel history was referred to the veterinary practice in Sobrance with unilateral conjunctivitis in the right eye. During the enquiry, the dog’s owner mentioned the administration of ofloxacin for 2 weeks with no clinical improvement. Consecutive ophthalmological examinations resulted in a discovery of a single thin white worm measuring over 10 mm in length. The worm was removed after application of a local anaesthetic and stored in formalin.

In the second case, an 8-year-old female hunting dog of the breed Wirehaired Slovakian Pointer, living in Hlivištia (48°48'19"N, 22°13'4"E), was presented to a private veterinary clinic in Michalovce with symptoms of bilateral conjunctivitis, epiphora and mucosae coating formations in a conjunctival sac. Ophthalmological examination revealed the presence of six thin white filiform worms in both eyes. After sedation the parasites were carefully removed using sterile tweezers and stored in 5% formalin. The dog was kept outside and has never left the country.

At the beginning of October 2016, two other cases were diagnosed in the same veterinary clinic in Michalovce. Unilocular conjunctivitis was diagnosed in a 5-year-old female Jack Russell Terrier from municipality of Michalovce, The dog was subjected to ocular examination, and after administration of anaesthetic drops, five eye-worms were removed from the conjunctival sac of the left eye and stored in 70% ethanol.

The second dog, a 4-year-old male Golden Retriever, lived in the village Vinné (also in the Michalovce district) (Fig. 1). After ophthalmological examination, four worms were collected from the right eye and preserved in ethanol.

### Morphological and molecular identification

All worms were delivered to the Institute of Parasitology, Slovak Academy of Sciences, Košice, for follow-up examination. All specimens (16 worms) preserved in formalin and/or ethanol were used for morphological examination using a Leica DM4000B microscope (Leica Microsystems GmbH, Wetzlar, Germany). Morphological measurements were performed and photographs were taken using a Leica DFC 290 HD camera and the software Leica Application Suite V 3.8.0 (Leica Microsystems GmbH, Wetzlar, Germany). The nematodes were identified according to morphological keys and previous descriptions [18, 19].

In order to confirm the morphological identification, the worms preserved in ethanol were used for molecular analyses. DNA was extracted using a commercial isolation set DNeasy Blood & Tissue Kit (Qiagen®, Hilden, Germany) according to the manufacturer’s instructions. PCR targeting of a fragment of 28S rRNA gene was performed using a set of universal nematode primers C1 (5'-ACC CGC TGA ATT TAA GCA T-3') and D2 (5'-TCC GTG TTT CAA GAC GG-3') [20]. Additionally, a portion of the cytochrome *c* oxidase subunit 1 (*cox*1) gene was amplified using primers NTF (5'-TGA TTG GTG GTT TTG GTA A-3') and NTR (5'-ATA AGT ACG AGT ATC AAT ATC-3') [21]. Both PCR reactions consisted of a polymerase activation step at 94 °C for 2 min, followed by 35 cycles of denaturation at 94 °C for 30 s, annealing at 55 °C for 30 s, and extension at 72 °C for 30 s, followed by a final extension at 72 °C for 7 min. After amplification, PCR products were visualized using 1.2% agarose gel electrophoresis.

 Consequently, amplicons were purified using NucleoSpin® Gel and a PCR Clean-up kit (Macherey-Nagel GmbH & Co., KG, Düren, Germany) and sequenced in both directions. Nucleotide sequences were manually edited and further compared with GenBank entries using BLAST (Basic Local Alignment Search Tool).

## Results

**Fig. 2:**
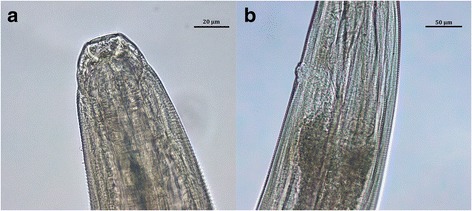
*Thelazia callipaeda* female. **a** Buccal capsule with a hexagonal opening. **b** Anterior position of the vulva in relation to oesophago-intestinal junction

**Fig. 3:**
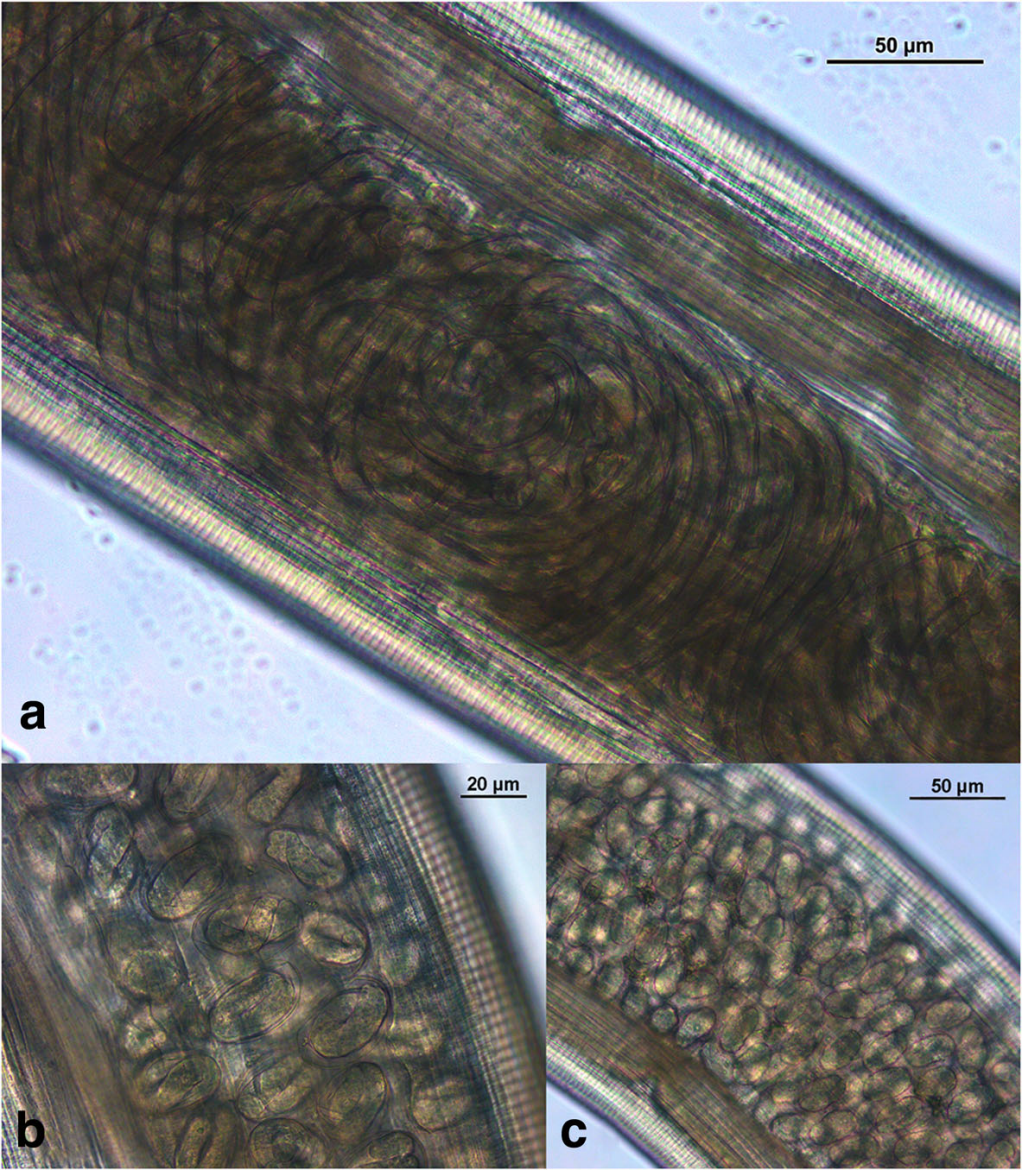
Uterus of a female *Thelazia callipaeda*. **a** Proximal area containing L1 larvae. **b** Middle area filled with embryonated eggs. **c** Distal area with immature eggs

Twelve of the worms recovered were adult females and four were adult males. Based on morphological features, anamnestic and clinical data, these worms were identified as *Thelazia callipaeda.* The average length of the female nematode body was 11.20 mm, and the body width varied between 336 and 391 μm. The average length of males was 8.99 mm and their width ranged between 318 and 455 μm. The worms exhibited a characteristic serrated cuticle with transverse striations, predominantly in the head area, and a hexagonal buccal capsule (Fig. 2a). The parasites were identified as *T. callipaeda* by the anterior position of the vulva in relation to the oesophago-intestinal junction (Fig. 2b), which serves as a distinguishing feature of *T. callipaeda* females. The distance between the vulva opening and the oesophago-intestinal junction reached on average 93 μm, and the distance between the vulva opening and the buccal extremity achieved an average of 261 μm. In all females, the uteri were filled with embryonated eggs. In one female worm a uterus was well defined to the proximal area containing L1 larvae (Fig. 3a), the middle area with embryonated eggs (Fig. 3b) and the distal area with immature eggs (Fig. 3c). The caudal end of *T. callipaeda* males was ventrally curved and showed the presence of pre-cloacal and post-cloacal papillae and dissimilar spicules. In addition, to confirm the identity of the species on the molecular level, PCR amplification of ribosomal and mitochondrial gene loci fragments was carried out. The BLAST analysis of a 645 bp long portion of the mitochondrial *cox*1 gene confirmed *T. callipaeda* as an etiological agent of infections. In accordance with the existence of low genetic variability within the European *T. callipaeda* population [2], alignment of an overlapping region of available *cox*1 nucleotide sequences demonstrated 100% identity of the sequence from Slovak dog with the h1 sequence haplotype isolated from Romanian dogs (KT716013, KP087796) and/or wild European rabbit (*Oryctolagus cuniculus*) from Portugal (KX033489). Nucleotide sequences of *T. callipaeda cox*1 and 28S rDNA gene fragments isolated from Slovak dog were submitted to the GenBank database under accession numbers KY476400 and KY476401, respectively.

## Discussion

The present study reports on the first cases of canine thelaziosis caused by *Thelazia callipaeda* in the territory of Slovakia. Considering that all presented cases were diagnosed in animals that had never travelled abroad, there is clear evidence of an autochthonous occurrence and thereby of the further spread of this nematode across Europe. While in the past decades canine thelaziosis has been commonly diagnosed in Italy and western European countries, during the last few years, new cases are being regularly reported from eastern Europe [10, 16]. The autochthonous infections presented here confirm the parasite’s establishment in a new area, and to the best of our knowledge, with latitude of 48°N, this could be considered as the most northerly located within western and central Europe. Regarding neighbouring countries, *T. callipaeda* was recently reporting in one dog from Hungary [16]. Additionally, one case of imported canine thelaziosis was described in the Czech Republic in a dog that came from the Lago Maggiore Region in northern Italy [22]. No cases have been reported from other neighbouring countries, i.e. Austria, Poland and the Ukraine.

In Europe, *T. callipaeda* is transmitted by the drosophilid species *Phortica variegata*; this has been confirmed under experimental and natural conditions [2, 3]. *Phortica* spp. fruit flies prefer forest habitats with a relative humidity ranging between 50 and 75%, while adults dwell in the tree canopies [23]. The occurrence of *P. variegata* has been described even in the eastern part of Slovakia, specifically along the Slovak-Ukraine border in Malé Trakany, Boťany and Stakčín [24]. The last mentioned municipality, Stakčín, is quite close to the sampling localities.

Two of the infected dogs live in the Sobrance District (Košice Region, eastern Slovakia) and the direct distance between the two localities (Koromľa and Hlivištia) is only 11.42 km (Fig. 1). The altitudes of the sites are 280 and 265 m, respectively. Sobrance is the easternmost district of the Košice Region, located on the border with the Ukraine, and bounded on the north by the Western Carpathians and on the south by the Eastern Slovak Lowland. This area is characterized predominantly by a rural environment. Several unique biotopes occur here, for instance the National Natural Reserve “Senné Ponds”, which is included in the global network of IBAs (Important Bird and Biodiversity Areas), or the “Zemplínska Šírava” Reservoir, covering an area of 33 km^2^, which represents the 12th largest water surface in Europe.

Both cases diagnosed in October 2016 were recorded in dogs from the adjacent Michalovce district. Again, the direct distance between both localities (Michalovce and Vinné) is very short, only 8.30 km. The village Vinné (altitude 148 m) is located only 3 km away from the above-mentioned “Zemplínska Šírava” Reservoir and belongs among the most popular touristic localities in eastern Slovakia. The artificial dam “Vinianske Lake”, with a total area of 8 ha, is situated in the village. Vinné is surrounded by dense deciduous forests to the north, and forest-steppe with thermophilic vegetation to the south.

The climate of the Sobrance and Michalovce districts is characterized as warm and moderately humid with a mild winter. The mean annual air temperature reaches 9–10 °C, with an average annual number of warm days (T_max_ ≥ 25 °C) of 60–70, average annual precipitation between 700 and 800 mm and average annual relative air humidity of 75–77.5% [25]. All of the above-mentioned factors, such as the habitat (forest vicinity), the climatic conditions as well as the dogs’ management (hunting dogs and/or dogs kept outside the whole year, with a significant amount of time repeatedly spent in a forest environment) could play in these cases an important role in the transmission of thelaziosis in this region.

## Conclusions

To our knowledge, the present study reports the first autochthonous cases of canine thelaziosis caused by *T. callipaeda* in Slovakia and provides clear evidence of the further spread of this parasite across Europe. Moreover, at the latitude 48°N, these cases might be considered as the northernmost autochthonous cases recorded within Europe. What is more relevant regarding the zoonotic potential of *T. callipaeda* and eventual spread is that, not only veterinarians but also human physicians, should be alerted to include thelaziosis as the possible cause of ocular infections in differential diagnosis.
